# Determination of Mineral Content in Methanolic Safflower (*Carthamus tinctorius* L.) Seed Extract and Its Effect on Osteoblast Markers

**DOI:** 10.3390/ijms10010292

**Published:** 2009-01-12

**Authors:** Young Seok Lee, Chang Won Choi, Jae Jin Kim, Andy Ganapathi, Rajangam Udayakumar, Sei Chang Kim

**Affiliations:** 1 Department of Biology & Medicinal Science, Pai Chai University, Daejeon 302-735, Korea. E-Mails: lysbone@empal.com (Y. L.); choicw@pcu.ac.kr (C. C.); jaejkim@pcu.ac.kr (J. K.); udayabiochem@yahoo.co.in (R. U.); 2 Department of Biotechnology, Bharathidasan University, Tiruchirapalli 620024, Tamilnadu, India. E-Mail: aganapathi2003@rediffmail.com

**Keywords:** Safflower (Carthamus tinctorius L.) seeds, methanolic extract, osteoblast activities, osteocalcin, bone-specific alkaline phosphates, IGF-I

## Abstract

Safflower (*Carthamus tinctorius* L.) seeds are used as a folk medicine to enhance bone formation or to prevent osteoporosis in Korea. Therefore, the methanolic extract of safflower seeds (MESS) containing high mineral content, such as calcium (Ca), potassium (K) and phosphorous (P), was evaluated for the role on osteoblast (Ob) markers of Sprague-Dawley rats. In serum of 3 to 11 weeks (wks) old rats, both osteocalcin (OC) content and bone-specific alkaline phosphatase (B-ALP) activity increased to their maximum levels in 4–7 wks. Hence, 3 wks old rats were selected for 8 wks oral treatment of MESS, resulted in the significant increase of Ob markers in serum such as OC content (4–8 wks), B-ALP activity (1–2 wks) and insulin-like growth factor I (IGF-I) level (1 wk), and the growth parameter such as the length of femur (2–8 wks) and tibia (4 wks). On the basis of Pearson’s correlation coefficient, there were a moderate correlation between OC and B-ALP at 8 wks, a low correlation between OC and IGF-I at 1, 4 and 8 wks, a moderate correlation between OC and femur length at 1, 2 and 8 wks, and a moderate correlations between OC and tibia length at 1 and 8 wks of MESS-treated groups. The result reveals that the changes of OC correlated at low to moderate level with the changes of B-ALP activity, IGF-I content and femur and tibia length in the MESS-treatment period. On the other hand, there were a strong correlation between IGF-I and femur length at 2 wks and moderate correlation between IGF-I and tibia length at 1, 2 and 8 wks of MESS-treated groups. Therefore, the effect of MESS on bone formation likely appears to be mediated by IGF-I at the early stage of treatment.

## 1. Introduction

Osteoblast (Ob) plays a critical role in bone formation through the proliferation and differentiation [1]. The latter is referred as obvious bone rigidity and strength with some degree of elasticity. Ob is the bone-forming cells responsible for the production of the bone matrix constituents, mainly type I collagen which becomes mineralized by deposition of Ca and non-collagenous protein. About 1% of the matrix is made up of osteocalcin (OC). OC plays a role in Ca binding and stabilization of hydroxyapatite in the matrix and/or regulation of bone formation [2]. Serum OC levels are higher in individuals undergoing rapid bone loss and lower in those with reduced bone turnover [3]. Bone-specific alkaline phosphatase (B-ALP) is a non-collagenous protein secreted by Ob, which is essential for bone mineralization [2]. Increased B-ALP levels in serum has been observed in conditions such as postmenopause, ovariectomy and osteoporosis in women [4], rapid bone loss [5] and fracture risk [6, 7]. OC and B-ALP levels in humans are relatively high during growth but lower after skeletal growth has brought to an end. Both levels also remain low in premenopausal women and middle-aged men, and then increase in older individuals including elderly men and post-menopausal women [8].

Serum insulin-like growth factor I (IGF-I) - a predominant growth factor produced by Ob is a determinant of bone size and mass in postnatal life [9]. IGF-I mediates the stimulatory effect of growth hormone (GH) on longitudinal bone growth [10] and also required for the anabolic actions of parathyroid hormone on bone [11]. Earlier studies demonstrated that animal dietary supplementation with dried plum, a rich source of phenolic and flavonoid compounds prevents the detrimental effects of ovarian hormone deficiency on bone density and trabecular structure [12] and reversed bone loss in the same model [13]. It has been suggested that the protection in ovarian hormone deficiency appears to be resulted from enhanced bone formation mediated through IGF-I [14]. In addition, the dried plum prevents osteopenia in androgen deficient male rats, and these improvements may be partly due to decrease in osteoclastogenesis via stimulation of bone formation mediated by IGF-I [15].

Safflower (*Carthamus tinctorius* L.) is a member of the family *Compositae* or *Asteraceae*. Its seeds contain a natural pigment known as carthamin derived from precarthamine by enzymatic reaction [16]. Traditionally, safflower has been used for purgative and alexipharmic effects, as well as in a medicinal oil to promote sweating and cure fevers in the Middle East, India and Africa [17]. In China, it is widely used in the treatment of many disorders and diseases including menstrual problems, cardiovascular disease, pain and swelling associated with trauma [18], chronic and atrophic gastritis [19], ankyloenteron [20], rheumatism [21], and chronic nephritis [22]. Especially, it has been used mainly for the treatment of cardiovascular disease because it invigorates the circulation and reduces blood cholesterol levels [18]. In Korea, the safflower seed extracts have traditionally been used for the treatment of blood stasis, the promotion of bone formation and the prevention of osteoporosis [23]. Safflower seeds might also have a potentiality to use as a drug for bone regeneration [24], Ob mineralization [25] and regeneration of periodontal defect [26]. In addition, safflower seeds have a protecting effect on bone loss caused by estrogen deficiency without substantial effect on the uterus [27]. A Korean herbal formulation, Gami-Honghwain, is comprised of crude ingredients from safflower seeds and hominis placenta. The Gami-Honghwain inhibited the elevated production of IL-1β which is implicated in the osteoporosis [28] and stimulation of the proliferation, differentiation and mineralization of osteoblastic MC3T3-E1 cells [29].

There are reports about chemical components of the safflower. Its leaves contain eight flavonoids, some of which showed potent antioxidant activities [30]. Its seeds also contain numerous polyphenolic compounds such as lignans, glucosides, flavonoids and serotonins. The chemical structures and antioxidant properties of these compounds have been characterized [31]. These compounds, termed phytoestrogens, are known to have weak estrogenic or antiestrogenic activity towards mammals [32]. However, no information is available on mineral content of safflower seeds. The aim of present study was to determine the mineral content in methanolic extract of safflower seeds (MESS) and examine its biological effects on rats’ Ob cells by the analysis of biochemical and morphological parameters. It will also provide the scientific basis for the usage of safflower seeds in the treatment of bone formation related with health.

## 2. Results and Discussion

### 2.1. Comparison of mineral content between aqueous and methanolic safflower seed extracts

Bone is a connective tissue mainly composed of organic and inorganic materials like collagen and the inorganic mineral hydroxyapatite, both of which combine to allow bone to be flexible and strong. The organic components include cells, as well as fibers which are similar to those that make up other connective tissues such as cartilage, while the inorganic minerals constitute predominantly Ca, P, CO_3_^2–^ and minor ingredients [33]. To determine the mineral content in safflower seeds, we used high-throughput ICP-MS, and this approach included the quantification of ten elements. In the aqueous extract from safflower seeds, there are K (2.306 μg/g), P (1.043 μg/g), Mg (0.474 μg/g), Al (0.175 μg/g), Fe (0.100 μg/g), Ca (0.075 μg/g), Zn (0.070 μg/g), Na (0.066 μg/g), Cu (0.055 μg/g) and Sr (0.022 μg/g). In the MESS, there are Ca (3.752 μg/g), K (1.313 μg/g), P (1.161 μg/g), Na (0.177 μg/g), Fe (0.170 μg/g), Zn (0.042 μg/g), Mg (0.023 μg/g), Al (0.019 μg/g), Cu (0.015 μg/g) and Sr (0.002 μg/g). It is observed that Ca, K and P are orderly high in methanolic extract, while K, P and Mg are orderly high in aqueous extract (Table 1).

Ca is necessary for many processes in the body, including contraction of muscles, nerve function, blood coagulation, and cell division. Only 1% of the Ca in the body is available in circulation for these functions, while the remaining 99% is housed in the bones. Osteoclast breaks down bone and converts the Ca salts into a soluble form which is easily circulated in the blood. Eventually, Ca salts are deposited on the surface of the bone matrix. If enough Ca is not available, bones cannot be properly mineralized [34]. K salts may benefit bone health by providing an anion that can be metabolized completely to carbon dioxide, or influence Ca excretion directly [35]. In addition, dietary K intake may exert a modest influence on markers of bone health, which over a lifetime may contribute to a reduced risk of osteoporosis [36]. Bone stores most P within the body to mineralize its matrix and serve as a mineral source. Inorganic phosphate plays a critical role in the maintenance of mineralized tissues and signaling in the intracellular environment [37]. In the present investigation, the MESS was selected for further analysis because it has high Ca, K and P contents.

### 2.2. Dependence of OC content and B-ALP activity on aging of rats

**Figure 1.**
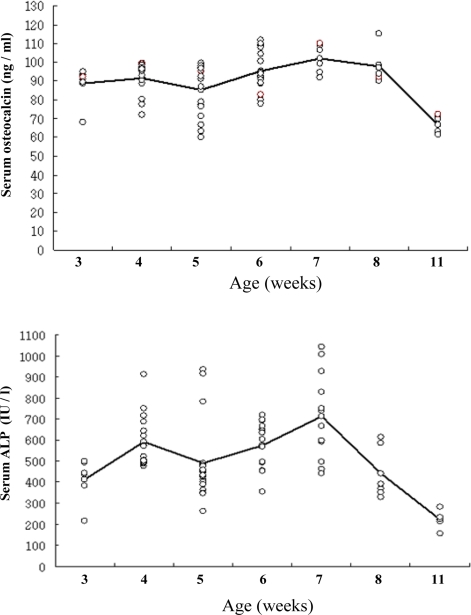
Changes of osteocalcin content (upper panel) and bone-specific alkaline phosphatase activity (lower panel) in serum of Sprague-Dawley rats by age groups. Mean of measurement values indicated on the line.

Although the precise function of OC and B-ALP are not clear, they are known as serum markers reflecting Ob activities including bone formation and turnover [38, 39]. Ob markers associated with aging, like OC content and B-ALP activity in serum of rats aged from 3 to 11 weeks (wks) were analyzed. Figure 1 illustrates the relationship between age and OC or B-ALP in rats. The serum OC content (ng/mL) of rats aged 3, 4, 5, 6, 7, 8 and 11 wks old were 88.85±3.54, 91.45±2.06, 85.27±3.41, 95.40±2.79, 101.97±3.13, 97.88±3.69 and 66.80±1.96. The B-ALP activities (IU/l) in rats aged 3, 4, 5, 6, 7, 8 and 11 wks old were 412.83±42.81, 590.88±30.97, 490.44±44.88, 575.82±16.62, 712.92±59.62, 440.33±51.39 and 221.0±16.57. The results showed the normal variation in OC and B-ALP levels of rats and also revealed the age influence on this variation. OC and B-ALP levels in serum of rats are positively correlated with each other, and both markers appeared as bimodal pattern. The 1^st^ peak was observed at 4 wks old and the 2^nd^ peak was at 7 wks of age. After this time, there was a steep drop in serum B-ALP activity from 7 to 11 wks and its reduction rate was approximately 67%. However, there was a slight decline in serum OC content from 7 to 11 wks of age and its reduction rate was approximately 30%. This tendency is consistent with previous reports in that the rats showed the highest B-ALP activity from 5 to 7 wks old [40] and the rapid decrease of B-ALP activity from 8 to 12 wks old [41].

### 2.3. Effects of methanolic safflower seed extracts on OC contents and B-ALP activities

**Figure 2.**
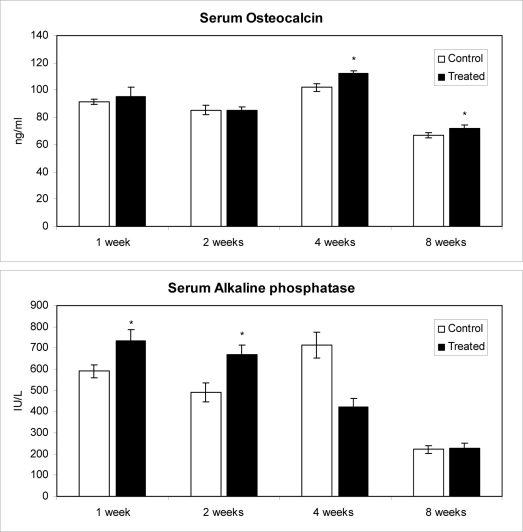
Concentration of osteocalcin and bone-specific alkaline phosphatase in serum after oral administration of *Carthamus tinctorius* extract. Values are mean ± S.E. *Statistically significant difference between experimental and control group by Student’s t-test at 0.05. C: control group and T: administration of *Carthamus tinctorius* was started at age of 3 wks old.

Compared with control rats, there were no significant differences in the increase of OC content of rats at 1 (4 wks old) and 2 (5 wks old) wks after oral treatment with MESS. However, there were significant differences (P < 0.05) at 4 (7 wks old) and 8 (11 wks old) wks after MESS treatment by the increase of 10.2% and 8.1%, respectively (Figure 2). Rats at 4 wks after MESS treatment showed the highest OC contents. These results suggest that the treatment with MESS induce rats’ Ob cells, thereby increase the secretion of OC from 4 to 8 wks after oral administration.

On the other hand, there were significant differences (P < 0.05) in B-ALP activity of rats administered orally with MESS at 1 (24.5%) and 2 (36.8%) wks but no significant differences at 4 and 8 wks after MESS treatment (Figure 2). The maximum B-ALP activity of rats treated with MESS appeared at 1 wk and the maximum percentage of increase appeared at 2 wks, while the peak activity of control rats appeared at 4 wks. Similarly, it has been reported that phenolic glycosides isolated from MESS increase the ALP activity of human Ob-like cells [42]. B-ALP is known as a biomarker for Ob differentiation and its upregulation occurs at the middle stage of differentiation [43]. In the present study, MESS stimulates Ob to synthesize of B-ALP at the early stage of treatment. However, we observed a low correlation between OC and B-ALP of MESS-treated groups at 1, 2 and 4 wks (r = 0.1054, r = 0.3015 and r = –0.3973, respectively) and a moderate correlation at 8 wks (r = –0.5955).

### 2.4. Effect of methanolic safflower seeds extract on serum IGF-I levels and long bone growth

**Figure 3.**
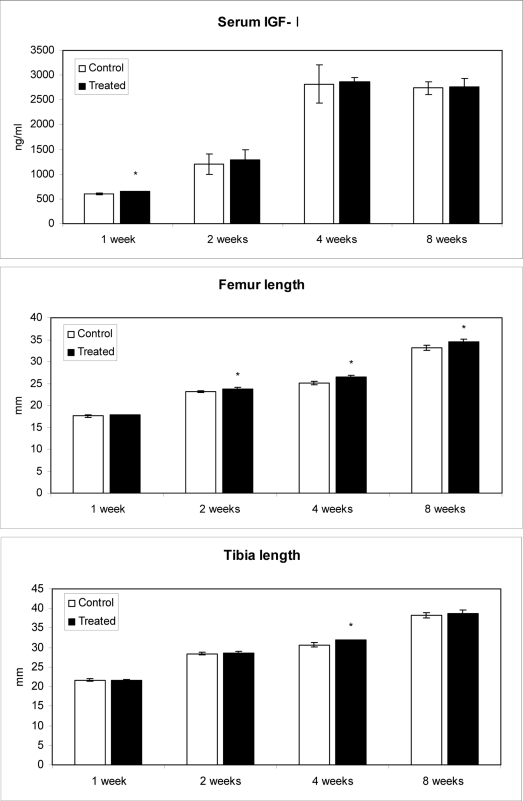
Concentration of IGF-I in serum and length of femur after oral administration of *Carthamus tinctorius* extract. Values are mean ± S.E. *Statistically significant difference between experimental and control group by Student’s t-test at 0.05. C: control group and T: administration of *Carthamus tinctorius* was started at age of 3 weeks old.

Serum IGF-I levels in both control and MESS-treated rats increased with age and reached the highest level at 4 wks during the experimental period. A significant increase of IGF-I level (7.52%) was observed at 1 wk after MESS treatment, and IGF-I was slightly higher in MESS-treated rats than in control throughout the experimental period (Figure 3). In earlier studies, IGF-I increased the number and proliferation of Ob cells, stimulated bone formation, maintained bone mass, and acted as a local proliferation and maturation factor for chondrocytes in the growth plate, which is essential for longitudinal bone growth [44]. IGF-I is abundant in liver and hepatic synthesis of IGF-I could account for the known turnover of this peptide in the circulation [45]. Biochemical markers of bone formation and bone resorption correlated with circulating GH and IGF-I levels, suggesting that GH and liver-derived IGF-I may have direct effects on Ob cell in modulating turnover [46]. Recently, the role of liver-derived IGF-I have been questioned in relation to effects on bone metabolism [47, 48]. The concentration of free IGF-I and -II in normal human serum is less than 0.5% of the total concentration of the IGF [49]. Despite the fact that this component is crucial in the feedback regulation of pituitary GH secretion and endocrine action of IGF-I on target tissues [50].

To determine MESS effect on the bone length, we measured the length of rats’ femurs and tibias at 1–8 wks after treatment. There were significant increases (P < 0.05) in femur length at 2 (3.04%), 4 (5.40%) and 8 (4.28%) wks after MESS administration (Figure 3). We observed a strong correlation between IGF-I and femur length of MESS-treated groups at 2 wks (r = 0.7005). However, there was a low correlation at 1 wk (r = 0.2852) and no correlation at 4 and 8 wks (r = 0.0031 and r = 0.0980, respectively). In addition, there was a significant increase (P < 0.05) in tibia length at 4 (4.07%) wks after MESS administration (Figure 3) but a low correlation between IGF-I and tibia length at 4 wks (r = 0. 1955). On the contrary, there was no significant increase in tibia length at 1, 2 and 8 wks after MESS administration but a moderate correlation between IGF-I and tibia length at 1, 2 and 8 wks (r = –0.6014, r = –0.5045 and r = 0.5000, respectively). In earlier studies, there is a positive correlation between serum IGF-I levels and bone mass in mice and humans [51, 52] and also bone mineral content, bone mineral density and femur length [9]. During the active bone growth period (3 to 11 wks), rats fed with MESS showed the elongation of the femur and tibia, which represents an effect on bone modeling (affects size and shape of whole bone and occurs of long time period, for example skeletal maturity about 18 years in human) but not bone remodeling. Therefore, the present study indicates that the effect of MESS on bone formation likely appears to be mediated by IGF-I at the early stage of treatment.

Moreover, during this period of time (bone modeling) Ob markers are expected to be increased, but we observed a low correlation between OC and IGF-I of MESS-treated groups at 1, 4 and 8 wks (r = 0.2059, 0.2043 and 0.1005, respectively) but not at 2 wks (r = – 0.0003). A moderate correlation between OC and femur length of MESS-treated groups was observed at 1, 2 and 8 wks (r = – 0.4998/ 0.5000 and– 0.5980, respectively) and a low correlation at 4 wks (r = – 0.1063). A moderate correlation was also observed between OC and tibia length for MESS treated groups at 1 and 8 wks (r = – 0.4010/– 0.4024), and a low correlation at 2 wks and 4 wks (r = – 0.2985/– 0.2994). Though we could not predict clearly through Pearson’s correlation coefficient, the percentage changes in OC content were not related to the changes of B-ALP activity, IGF-I content and femur and tibia length. However, the changes of OC correlated at low and moderate levels to the changes of B-ALP activity, IGF-I content and femur and tibia length during the experimental period.

## 3. Experimental Section

### 3.1. Preparation of safflower seeds extract and analysis of mineral content

**Figure 4.**
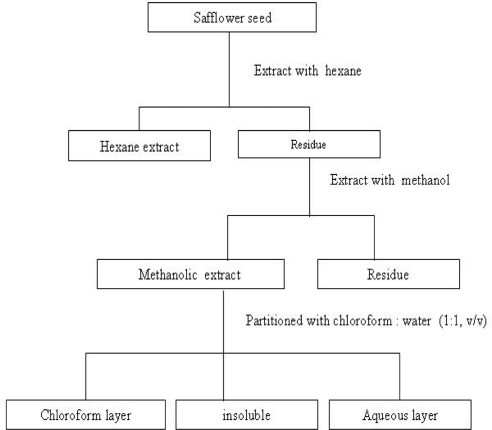
Flow chart showing the methanolic extraction from safflower seeds.

Safflower seeds were collected from a local medicinal market in Daejeon, Korea and homogenized by a crusher to obtain a crude powder, 300 g of which was extracted as described in Figure 4. In the final step of methanolic extraction, the aqueous methanol layer was evaporated under reduced pressure using a rotary evaporator at 40–50° C and freeze-dried as a powder. Another 300 g of powder was decocted in a round flask with distilled water (1:10, w/v) at 60° C for 3 h, then filtered using No. 40 Whatman filter paper to remove particulate matter and evaporated in a rotary evaporator under the same conditions mentioned above. The yields of crude methanol and water extracts were about 30 and 25 g, respectively. Each extract was heated at 55 ° C for 20 h, and then dissolved in 6 N HCl containing 1% LiCl_3_ to determine mineral content using an inductively coupled plasma mass spectrophotometer (ICP-MS Elan 6000, Perkin-Elmer, USA) as described in the manufacturer’s directions.

### 3.2. Experimental animals and administration of extract

Three wks old male Sprague-Dawley rats weighing 34.2±2 g were selected and housed in polycarbonate cages under a 12 h light/12 h dark cycle at a regulated temperature of 25±1° C and 55±5% humidity. A commercial standard pellet diet and water was available *ad libitum* throughout the experimental period. To compare OC contents, B-ALP activities and IGF-I contents between non-treated control group and treated group, six rats were used and administered once a day orally with MESS at a dose of 350 mg/kg body weight using an oral sonde (Natusme Seisakusho, Tokyo, Japan) for 8 wks. The dose was based on previous a report in that no toxic effect was observed from serum and urine indexes after feeding the safflower seed diet [53]. At the end of the oral administration period 1–8 wks (corresponding age 4–11 wks old), the rats were fasted for 12 h and then immediately sacrificed using ether. After confirming the deep anesthesia, heart puncture and orbital bleeding were performed and blood was collected from the main artery. Serum was separated by centrifugation at 3,000 rpm for 15 min. The schedules and procedures were performed in the experimental animal handling facility at the Department of Biology & Medicinal Science, Pai Chai University, in compliance with ethical regulations.

### 3.3. Biochemical analysis of serum

Serum OC content was determined using a Rat Osteocalcin EIA kit (Bioquote Limited, UK) as described in the manufacturer’s directions. Two OC antibodies were employed, each directed toward the *N* or *C*-terminal OC molecule. B-ALP activity in serum was determined using a commercially available ELISA kit (Alkphase B^®^, Metra Biosystema Inc., CA, USA). Serum IGF-I was measured by a double antibody immunoradiometric assay (IRMA) using Non Extraction IGF-I Bridge Kit (Adaltis, Italy) as described in manufacturer’s directions. The first antibody is immobilized to the inside wall of the tubes (anti-IGF-I-coated tubes) and the second antibody is radiolabelled for detection [anti-IGF-I- (I^125^)]. The radioactivity was measured in a gamma counter for 1 min and determined the net counts per min (CPM).

### 3.4. Measurement of long bone growth

To determine long bone growth, we used x-rays. The length of femurs and tibias in all treatment groups was measured three times to 0.01 cm using a calibrated digital Vernier caliper.

### 3.5. Statistical analysis

Data were expressed as the mean ± SE calculated from the specified numbers of determination and analyzed with SPSS 15.0 (SPSS, USA) to determine the significance of effect of safflower seed extract on OC, B-ALP, IGF-I and bone growth. Data comparisons were made by the Student’s t-test. Pearson’s correlation coefficients were used to test the association of OC with other parameters like B-ALP activity and IGF-I content in serum and femur and tibia length. All values less than 0.05 (p<0.05) were considered statistically significant.

## 4. Conclusions

MESS contains a high mineral content, such as Ca, K and P. Notable elevation was observed on IGF-I level at 1 wk, followed by a significant and maximum increase of B-ALP at 2 wks and OC at 4 wks after the administration of MESS into rats. The significant elongation of the femur was observed at 2, 4 and 8 wks after treatment with MESS, suggesting that the MESS effect on bone formation likely appear to be mediated by IGF-I at the early stage of treatment. However, OC content change might be less associated with the changes of B-ALP activity, IGF-I content, and femur and tibia length. In addition, MESS has no cytotoxicity under this experimental condition, hence, it can be used as a potential alternative to enhance bone formation.

## Figures and Tables

**Table 1.** Mineral contents of extracts from *Carthamus tinctorius.*

Elements	Contents (μg/g)	
	W^1)^	M^2)^
Al	0.175	0.019
Ca	0.075	3.752
Cu	0.055	0.015
Fe	0.100	0.170
K	2.306	1.313
Mg	0.474	0.023
Na	0.066	0.177
P	1.043	1.161
Sr	0.002	0.002
Zn	0.070	0.042

W^1)^ = Distilled water extract of *Carthamus tinctorius*.

M^2)^ = Methanol extract of *Carthamus tinctorius*.
